# HPV Type 6 and 18 Coinfection in a Case of Adult-Onset Laryngeal Papillomatosis: Immunization with Gardasil

**DOI:** 10.1155/2015/916023

**Published:** 2015-11-29

**Authors:** Virginia Fancello, Andrea Melis, Andrea Fausto Piana, Paolo Castiglia, Andrea Cossu, Giovanni Sotgiu, Corrado Bozzo, Emma Victoria King, Francesco Meloni

**Affiliations:** ^1^Division of Otorhinolaryngology, Department of Surgical, Microsurgical and Medical Sciences, University of Sassari, Viale San Pietro 43, 07100 Sassari, Italy; ^2^Hygiene and Preventive Medicine, Department of Biomedical Sciences, University of Sassari, Sassari, Italy; ^3^ENT Department, P. Dettori Hospital, Tempio Pausania, Italy; ^4^Department of Head and Neck Surgery, Poole Hospital NHS Foundation Trust, Poole, UK

## Abstract

Laryngeal papillomatosis (LP) is a rare human papillomavirus (HPV) related disease that often requires multiple surgical interventions and residual impairment of voice is almost inevitable. We report the case of a patient with adult onset recurrent LP, showing moderate dysplasia and coinfection with HPV types 6 and 18. The tetravalent HPV vaccine Gardasil was prescribed off label, with the aim of triggering an immunogenic response and consequently reducing the probability of further recurrences. The patient was followed for 9 months with no sign of relapse of his LP. The postexposure use of the anti-HPV vaccine could represent a promising therapeutic agent in established LP. Unfortunately, the potential efficacy of this new therapeutic option in this situation has been suggested only by isolated case reports. Further controlled studies, with a longer follow-up and a larger sample size, are needed to assess efficacy of Gardasil in LP.

## 1. Introduction

Laryngeal papillomatosis (LP) is a rare disease with estimated incidence in USA of 4,3/100 000 in children and 1.8/100 000 in adults [1], characterized by exophytic, wart-like lesions due to oral infection with human papilloma virus (HPV) type 6 or 11.

In contrast to the low incidence of LP, many studies have shown that HPV DNA can be detected in the upper airway of healthy adults and children [2], suggesting that the exposure to the virus happens frequently during life, almost comparable to other viruses such as rhinovirus. Therefore other factors must contribute to the development of persistent papillomatosis disease, such as a deficiency of an immune response [3].

In children, the infection is generally considered as vertically sexually transmitted from mother to child in the birth canal [4]. In adults, it is thought to be reactivation of a latent HPV infection, potentially acquired at birth, but the mechanism underlying the progression from HPV infection to LP remains unknown. The transmission may also occur during oral sex, but this has not been demonstrated [5].

Despite its benign nature, LP may significantly affect quality of life as it has a tendency to grow and extend throughout the entire respiratory tract, leading to dysphonia (voice alteration) and dyspnoea (respiratory pattern alteration). In addition, the role of HPV in malignant transformation is well-described and discussed [6].

For all these reasons, LP is a frustrating and a challenging disease to manage, which may require multiple surgical interventions to remove papillomas and residual impairment of voice is almost inevitable.

The ideal therapies aim to maintain airway patency, improve voice quality, and avoid complication.

Surgery, with classic cold knife microsurgery, laser (CO_2_, argon, and Nd-YAG), and microdebrider, is the preferred mode of treatment but does not prevent lesions from recurring.

Currently, no disease-specific medical therapy exists for LP: several treatments are used, but none can be considered predictive of cure.

Many drugs have been tried as adjuvant treatment including interferon alpha and injected or inhaled cidofovir. However, they are not universally accepted [7, 8]. Bevacizumab, indole-3-carbinol, photodynamic therapy, and cis-retinoic acid have also been tried, but without any proven efficacy [9].

Since 2006 two safe and highly immunogenic prophylactic HPV vaccines able to stimulate both humoral and cellular immunity, Gardasil (also known as Silgard), product from Merck, and Cervarix, product from GlaxoSmithKline, are licensed in more than 100 countries and immunization programs in adolescent girls have been widely diffuse with the intent to prevent cervical cancer [10, 11].

Gardasil is a quadrivalent vaccine made up of recombinant HPV proteins (L1 capsid antigens) from the most common high-risk HPV types (16/18), responsible for 70% of cervical cancer cases and HPV related head and neck cancer, as well as two low-risk HPV types (6/11) which are the causative agents for laryngeal papillomatosis and genital warts. Although the tetravalent vaccine is used to prevent infection by the four subtypes, it has also been used as adjuvant therapy to downregulate the disease in patients with LP.

Cervarix is a bivalent vaccine that was FDA approved three years after Gardasil, effective against HPV-16/18. In addition it seems to offer a cross-protection against types 31, 33, and 45, extending its action against 85% oncogenic types.

We present a case report of adult onset of LP treated with surgery and immunotherapy based on vaccination with Gardasil.

## 2. Case Report

We report on the case of a 48-year-old man, with no relevant comorbidities and no history of smoking or alcohol abuse, sexual transmitted infections, or immune deficiencies.

In his early forties he developed progressive hoarseness and at the age of 45 he was finally diagnosed with LP. According to the anamnesis data since the diagnosis three surgical interventions were performed: the first one with cold steel and the last ones with CO_2_ laser. After the final surgery an adjuvant treatment with multiple local injections of cidofovir was carried out. This was classified as nonaggressive according to the Lindeberg classification (less than ten operations in 1 year) [12]. Histopathology showed papillomatosis with focal signs of low grade dysplasia and immunohistochemistry staining positive for p16.

Following a new severe recurrence, characterized by confluent papillomas, he was admitted to the ENT Department, AOU SS, University of Sassari, Italy. On clinical examination the papillomatosis involved the epiglottis and the false and the true vocal cords bilaterally, resulting in mild dyspnea and severe dysphonia.

Surgical debulking was performed using CO_2_ laser. The histology reported papillomatosis with moderate and diffuse dysplasia. Tumor cells exhibited strong and diffuse nuclear and cytoplasmic stains for p16 (NK4a).

The virological analysis was carried out in triplicate at the WHO quality-assured laboratory of the Hygiene and Preventive Medicine Unit, Department of Biomedical Sciences, University of Sassari (http://www.who.int/biologicals/areas/vaccines/hpv_labnet/en/webcite).

A sample of formalin-fixed and paraffin-embedded tissue lesion was selected. DNA was isolated after extraction and purification using Easy-DNA kit [13]. HPV detection and genotyping were performed according to the manufacturer's instructions using the Anyplex II HPV-28 and a CFX96 real-time thermocycler (Bio-Rad, Hercules, CA, USA). The L1 gene of HPV and human beta-globin was simultaneously coamplified as an internal control to monitor DNA purification efficiency, PCR inhibition, and cell adequacy.

Coinfection by HPV-6 and HPV-18 was detected. Confirmation was obtained by testing the sample with an “in-house” real-time quantitative TaqMan PCR assay [14].

On the basis of the virological results, three biological samples, collected during the previous surgical interventions, were retrieved and analyzed using the above-mentioned molecular technique. All three were positive for HPV-6.

Until now in our department the first line treatment has always been surgical, with CO_2_ laser. In the past cidofovir has been considered an option but the risk of malignant transformation induced by this drug made it not suitable as adjuvant therapy. In addition to this last observation, considering also the failure of the previous attempts to improve the course of disease with antiviral medication, the detection of a viral coinfection with an oncogenic HPV subtype, and the increase in grade of dysplasia over the years, we chose to vaccinate the patient to try to stimulate an immunological response. Moreover the patient showed a large larynx involvement, the most extensive treated in our hospital in the last two decades, inducing us to believe in a necessity to find a good adjuvant therapy to gain a better outcome.

The patient was also tested for anti-HPV antibodies, without positive result, reinforcing the idea that laryngeal HPV infection is not always capable of triggering an adequate immune response [15]. However it should be clear that the interpretation of immunological status in patient with HPV infection has always been arduous. Patients can show a positivity of AB-HPV on serum even if the infection is cleared, and on the other hand patients with persistent HPV infection (e.g., women with cervical involvement) tend to show high HPV levels. Despite this data, in case of laryngeal papillomatosis with adult onset we frequently assist in an absence of antibodies compared with other kinds of HPV infection [3].

The consequent lack of correlation between antibodies level and laryngeal status suggests an inability to produce immune response. Therefore that evidence conferred a little value to the serology test as a screening investigation to detect high risk groups, because even when there is a correspondence with HPV DNA detection, it does not provide any proof of anti-HPV T cell activity, which plays a fundamental role in the immune response against the virus.

Considering all of this data the tetravalent HPV vaccine Gardasil was prescribed off label, with the aim of triggering a highly immunogenic response to HPV-6 and HPV-18 and consequently reducing the probability of further recurrences and malignant transformation. A three-dose schedule of vaccination in 1, 3, and 6 months was planned, with the first dose administered 1 month after the surgical intervention.

Since the surgical treatment and over a follow-up period of 12 months, no signs of relapse or other mucosal lesions were noted. This represents the longest documented period free of recurrence of our patient.

## 3. Conclusion

HPV vaccination, currently recommended for noninfected individuals, could represent a promising therapeutic agent in established LP. Unfortunately, the potential efficacy of this adjuvant therapeutic approach has not been assessed in multicenter clinical trials but has been implicated only by few case reports [16–19].

Only Boltezar et al. [20] and Young et al. [21] report on a larger cohort of patients: both studies show a positive correlation between Gardasil and better outcome in course of LP, as well as the isolated case reports. But the peculiar course of LP, characterized by spontaneous remissions and relapses, makes it difficult to demonstrate a direct relationship between adjuvant drugs and improvement of disease course.

We report a case of adult onset recurrent disease LP. Although nonaggressive, the management of the case was complicated by the coinfection of low risk HPV-6 with oncogenic HPV-18 and thus the risk of malignant transformation, as described in the literature [22, 23].

During 12 months of regular ENT examination, no sign of recurrence or sinister mucosal changes were observed, but a lifelong surveillance should be carried out to detect any early relapses or malignant transformation.

More extended studies with longer follow-up and a larger sample size are needed to assess the effectiveness of vaccination in patient with recurrent LP.

Another important consideration about the possibility to offer the vaccination in the patients with LP should be suggested by the evidence that this subgroup has less ability compared with the general population to clear this viral infection. Their susceptibility to contract HPV infection in lifetime makes them more exposed to the risk of HPV related cancer, as oropharyngeal cancer, whose rate showed increase in the last decades.

From this point of view LP subjects can be considered high risk group, reinforcing the utility of vaccination among them and suggesting a potential benefit from administration of both vaccines.

Hopefully the broad spread of HPV vaccines will offer in the future a further decrease of incidence of this disease.
